# Socioeconomic Barriers to COVID-19 Booster Vaccination in Southern Italy: A Retrospective Study to Evaluate Association with the Social and Material Vulnerability Index in Apulia

**DOI:** 10.3390/vaccines13121255

**Published:** 2025-12-18

**Authors:** Nicola Bartolomeo, Letizia Lorusso, Niccolò Maldera, Paolo Trerotoli

**Affiliations:** 1 Department of Interdisciplinary Medicine, Section of Medical Statistics, University of Bari Aldo Moro, Piazza Giulio Cesare 11, 70124 Bari, Italy; nicola.bartolomeo@uniba.it (N.B.); n.maldera4@studenti.uniba.it (N.M.); 2 School of Medical Statistics and Biometry, Department of Interdisciplinary Medicine, Section of Medical Statistics, University of Bari Aldo Moro, Piazza Giulio Cesare 11, 70124 Bari, Italy; l.lorusso80@studenti.uniba.it

**Keywords:** vaccine hesitancy, vaccination coverage, social setting, social deprivation, sex difference, public health, Social and Material Vulnerability Index

## Abstract

**Background**: Socioeconomic disparities may affect COVID-19 booster vaccination uptake, potentially undermining public health efforts. This study assessed the association between first booster dose coverage and municipal socioeconomic deprivation in the Apulia region of southern Italy. A secondary objective was to evaluate whether SARS-CoV-2 incidence modified this relationship. **Methods**: We conducted a retrospective observational study including Apulian residents aged ≥5 years from 1 January 2021, to 31 December 2022. First booster doses were identified using an algorithm based on dose chronology and national guidelines. Vaccination and infection data were retrieved from regional databases. Socioeconomic deprivation was measured using the Social and Material Vulnerability Index (SMVI) developed by the Italian National Institute of Statistics (ISTAT). Booster coverage was calculated at the municipal level. A multivariable Poisson’s regression model was used to estimate the association between SMVI and booster uptake, adjusting for age group, primary vaccine type, SARS-CoV-2 incidence, and municipal vaccination rates. Analyses were stratified by sex. **Results**: A total of 2,732,258 individuals received a first booster dose. Booster coverage decreased with increasing SMVI. Among females, a significant reduction was observed in the highest deprivation category (RR > 102 vs. <99: 0.95; 95% CI: 0.94–0.97) and it was similar in males (RR: 0.95; 95% CI: 0.93–0.96). A significant interaction between age and deprivation was found in both sexes, with a sharper decline in younger individuals. Municipal vaccination rates were positively associated with booster uptake. SARS-CoV-2 incidence was positively associated with uptake only in males. **Conclusions**: The analysis revealed a significant association between lower socio-cultural level and lower adherence to the first booster dose of the COVID-19 vaccine. The decline is more pronounced among subjects younger than 50 years with high levels of vulnerability. The findings of this study suggest that to overthrow vaccine hesitancy, knowledge of the social setting allows for targeted communications to the different groups in the population. Further research is needed to define different approaches in the different social groups.

## 1. Introduction

Since December 2019, the number of confirmed COVID-19 cases reported to the World Health Organization (WHO) has risen worldwide [1]. Up to this point, the WHO has systematically monitored the progression of cases. On 30 January 2020, the WHO declared the COVID-19 outbreak a Public Health Emergency of International Concern. Between February and May 2020, Italy underwent a nationwide lockdown due to the absence of a vaccine or other effective containment measures [2]. As reported in several studies, adherence to vaccination varied among countries but contributed to a reduction in COVID-19-related mortality, as noted by Chen, who reported a rate of eight doses per 100 people [3]. A U.S.A. population study evaluated the effectiveness of COVID-19 vaccination, showing that herd immunity could be achieved earlier with a faster vaccination pace, lower vaccine hesitancy, and higher vaccine effectiveness [4]. Chen (2023) identified three distinct vaccination patterns [3]. The full-speed vaccination pattern led to the greatest reduction in the spread of COVID-19 compared with the other two approaches. In the U.S.A., this pattern confirmed that the availability of an effective vaccine significantly modified the course of the SARS-CoV-2 pandemic. In other countries, such as Italy, corresponding to the Type C pattern proposed by Chen, the gradual increase in vaccination rates over the period was associated only with a reduction in COVID-19-related hospitalizations [3,5]. During the initial phase, high adherence provided protection against infection, also benefiting from herd immunity. Subsequently, the reduction in cases led to a decline in vaccination uptake, despite the increased risk of infection and severe COVID-19-related hospitalizations among the unvaccinated [5,6]. The same trend was confirmed by Havers et al. (2022), who collected data from 1 January 2021, to 30 April 2022 [7].

The reduction in perceived risk due to declining SARS-CoV-2 incidence, together with concerns about receiving a medical intervention while feeling healthy and the perception of an increased likelihood of adverse events, has led to a lower vaccine acceptance of COVID-19 vaccines [8,9].

Misinformation and cognitive biases, with a decline in trust for science, have exacerbated the phenomenon of vaccine refusal, with the support of associations that indicate vaccines as health hazards [10,11,12,13,14,15].

At the same time, vaccine refusal also leads to a reduction in herd immunity, so vaccination campaigns risk not achieving the desired effect of reducing morbidity and mortality [16,17].

The possible relationship between socio-cultural and economic conditions and vaccine refusal was already analyzed, even before COVID-19, in a study in 2018 [18] that analyzed the reason for pediatric vaccine refusal and used sociodemographic variables, such as employment status and educational level, and the odds of refusal that resulted were higher for unemployed participants, while they were not statistically significant for those with a higher educational level. A study conducted on vaccination coverage from 2006 to 2016 in Italy to evaluate the effect of misinformation and fake news on vaccination rates showed that the following hesitancy in vaccination in the population could have also been caused by the news about a court rule that decided to compensate damage for vaccine-related injury, while the statistical adjustment for socioeconomic factors in the model did not result significant in the refusal trend [19]. The systematic review by Truong et al. has concluded that lower income and lower educational level were the social characteristics more frequently associated with low vaccine acceptance [20]. Tiruneh et al. examined sociodemographic and health-related predictors of COVID-19 booster uptake among fully vaccinated adults in Texas, confirming significant disparities across geographic regions, racial and ethnic groups, and insurance status [21]. These findings highlight the need for targeted educational initiatives to promote booster vaccination among underserved populations. Building on this evidence, previous work by Langmuir et al. has shown that multiple approaches have been implemented in Canada to support behavior change and address the barriers and facilitators influencing COVID-19 vaccine uptake among priority groups [22].

The trust in those who proved the information and the medium that conveys the information should be considered relevant factors for the decision to accept vaccination, and ethnicity, occupation, and political beliefs are important factors for the interpretation of the messages and the decision to accept or reject the vaccination [23].

In Italy, socioeconomic determinants of vaccine hesitancy and refusal have been investigated across different population groups, including healthcare workers [24], students, and university communities [25]. Among students, hesitancy was more common in those reporting a poorer health status after the primary vaccination series, lacking friends or family diagnosed with COVID-19, or not receiving information from official government sources. Overall, as highlighted by the Italian National Institute of Health (ISS), COVID-19 vaccination acceptance or hesitancy in Italy is mostly influenced by the perceived safety, efficacy, and usefulness of the vaccine [26]. The objective of health managers to have a high rate of vaccination coverage, given what studies on vaccination have shown, requires knowledge of the social condition of the population in order to manage information and avoid mistrust and misinformation. In Italy, the Social and Material Vulnerability Index (SMVI), developed by the National Institute of Statistics (ISTAT) [27], summarizes socioeconomic deprivation through multiple dimensions—educational attainment, labor market participation, housing crowding, family composition, and material hardship—standardized at the municipal level. These structural determinants are known to influence access to health information, healthcare utilization, and trust in public health institutions, making the SMVI particularly suitable for examining patterns of COVID-19 booster uptake. On the other hand the availability of data and instruments to map the rate of vaccination could help to increase the status of the population to move towards and attempt to target information that improves the vaccination rates of the area [28].

In a preliminary analysis presented at the 13th Scientific Meeting of the Italian Society of Clinical Epidemiology and Medical Statistic, we have explored the association between SMVI and first booster dose, and the agreement between SVMI and the Caranci Deprivation Index; we observed that an association analysis could be performed with a better definition of the model and using only the SVMI [22].

The aim of this study is to evaluate the association between the rate of first booster dose of vaccine against SARS-CoV-2 and social inequalities, in order to evaluate the usefulness for public health officers managing aggregated data using maps and available data from different sources to decide the strategy of vaccination campaigns. We explored socioeconomic inequalities measured by the SMVI and rate of the first booster dose of the COVID-19 vaccine in the Apulia region and whether the incidence rates of SARS-CoV-2 infection influenced this association.

## 2. Materials and Methods

### 2.1. Study Context and Definition of the First Booster Dose

Apulia is a region in southern Italy with about 3,922,941 inhabitants in 2021, including 1.6 million families. It is the seventh largest Italian region (19,000 km^2^) with a population density of about 200 inhabitants/km^2^. In recent decades, Apulia’s economy has grown more than the rest of southern Italy, although per capita GDP in 2020 remained 36.3% below the national average.

Italy’s COVID-19 vaccination campaign began on 27 December 2020, initially targeting healthcare workers, nursing home residents, and high-risk groups, and progressively extended to the general population by mid-2021.

The booster dose is defined as the first “recall” or “reinforcement” dose to be administered at the end of the primary vaccination cycle (comprising two doses for Pfizer’s Comirnaty, Moderna’s Spikevax, and AstraZeneca’s Vaxzevria vaccines, and a single dose for J&J’s COVID-19 Vaccine Janssen), after a specified interval of time. The first booster dose campaign was launched on 27 September 2021 [29], initially requiring a 6-month interval after the primary cycle, later reduced to 5 months [30] and then to 4 months [31] in response to the spread of the Omicron variant. The dataset on COVID-19 vaccinations did not include a specific variable identifying booster doses. For each individual, vaccination histories were reconstructed, and the first booster dose was defined using a standardized algorithm based on chronological order, vaccine type, and national guidelines. A detailed description of this procedure is provided in the Supplementary Materials (“Algorithm for identifying the First Booster Dose” paragraph and Supplementary Figure S1).

### 2.2. Study Design and Data Sources

We conducted a retrospective ecological study using municipal-level data for all residents of the Apulia region (southeastern Italy) aged ≥5 years between 1 January 2021, and 31 December 2022. Individual vaccination records were used to identify and classify first booster administrations. Municipal first booster dose rates (FBDRs) were then calculated using the entire resident population aged ≥5 years as the denominator, and their association with a municipal-level indicator of socioeconomic vulnerability provided by the National Institute of Statistics (ISTAT) [27] was assessed. The municipal incidence rate of SARS-CoV-2 infection was included as an adjustment variable to explore whether local infection dynamics influenced the association between deprivation and booster coverage.

Data on COVID-19 vaccinations were retrieved from the Regional Immunization Information System (GIAVA), which collects information on sex, age, municipality of residence, vaccine type, administration date, and dose number. Cases of SARS-CoV-2 infection were obtained from the regional IRIS (Infections Regional Information System) surveillance platform, implemented using the WHO Go.Data tool (Version 2, 18 March 2024) [32]. Demographic information on the resident population was obtained from the ISTAT Demo database, stratified by sex and age [33]. Children aged 0–4 years were excluded, as booster doses were not recommended for this group during the study period. The variables used in the analysis (municipality, age, and sex) are mandatory fields in the regional vaccination registry; therefore, no missing data were present. Occasional inconsistencies in the chronological order of dose labels were automatically resolved by the booster-identification algorithm, which incorporates both dose numbering and temporal intervals between administrations. For this study all data were managed as aggregated counts by city, age, and sex so that it was no longer possible to trace it back to an individual, so authorization from an ethics committee is generally not required. This type of observational study was in the categories listed by the Regulation of 8 August 2024 as not being subject to ethical committee review.

### 2.3. Socioeconomic Deprivation

Socioeconomic deprivation at the municipal level was assessed using the Social and Material Vulnerability Index (SMVI) developed by ISTAT. The SMVI is a composite indicator based on seven dimensions:

1. Incidence of single-parent households (young < 35 years, or adult 35–64 years).
2. Incidence of households with six or more members.
3. Proportion of individuals aged 25–64 years with no educational qualification or illiteracy.
4. Incidence of households composed only of elderly members (≥65 years) including at least one aged ≥80 years.
5. Severe overcrowding, defined as households with more occupants than allowed by dwelling size.
6. Proportion of young people (15–29 years) not in education, employment, or training (NEET).
7. Incidence of households with children in which no adult is employed or retired from previous employment.

Raw indicators were normalized to account for variability and to allow comparisons over time, with a reference value of 100 corresponding to the national mean in 1991; higher SMVI values indicate higher vulnerability. For the purposes of this analysis, municipalities were categorized into five classes based on the quintiles of the distribution of SMVI: very low (VL), SMVI less than 99.3; low (L), SMVI between 99.3 and 100.1; medium (M), SMVI between 100.1 and 101.0; high (H), SMVI between 101.0 and 101.9; and very high (VH), SMVI greater than 101.9. This choice reflects the typical use of deprivation indices in epidemiological studies and rests on the assumption that, although continuous deprivation scores may fluctuate, shifts between quartiles are less frequent and therefore provide a more stable proxy of socioeconomic position in longitudinal analyses [27].

### 2.4. Statistical Analysis

The unit of analysis was the municipality (n = 257). All vaccination and infection data were aggregated at the municipal level before modeling. The first booster dose rate (FBDR) was calculated as the number of first booster doses administered divided by the resident population aged ≥5 years, expressed as a percentage. Descriptive analyses were performed by sex, age grouped into nine classes (5–19, 20–29, 30-39, 40–49, 50–59, 60–69, 60–79, and 80+ years), and SMVI class.

To assess associations between FBDR and socioeconomic deprivation, we first fitted Poisson’s regression models. Evidence of overdispersion was observed. Negative binomial models were therefore adopted, which provided unbiased standard errors under heterogeneous count variability and better fit based on AIC and BIC values. The model included the following covariates, selected a priori based on established determinants of COVID-19 vaccination uptake:

- Age group; reference: ≥80 years.
- Socioeconomic deprivation (SMVI) categorized into quartiles to reflect typical epidemiologic use; reference: lowest deprivation (<99).
- Municipal SARS-CoV-2 infection rate, included as a context-level determinant of risk perception.
- Vaccination coverage rate (≥1 dose), included as a marker of local vaccination behavior and access.
- First dose viral vector vaccine rate, to account for early-phase vaccine allocation differences across municipalities.

Because socioeconomic effects may differ by age, we also included the age × SMVI interaction, which significantly improved model fit.

Separate models were fitted for females and males.

Results are reported as first booster dose rate (FBDR), rate ratios (RRs), and their 95% confidence intervals (CIs). Pairwise comparisons were adjusted by Tukey’s test. Statistical significance was set at *p* < 0.05 (two-sided). As a sensitivity analysis, we repeated all models, stratifying municipalities into three groups (<15,000, 15,000–50,000, ≥50,000 inhabitants) to assess potential ecological bias related to the use of SMVI at the municipal level.

All analyses were performed using SAS software, version 9.4 (SAS Institute Inc., Cary, NC, USA).

## 3. Results

Between 1 January 2021, and 31 December 2022, a total of 3,472,095 residents in Apulia aged 5–110 years received at least one COVID-19 vaccine dose, of whom 51.6% were female. Overall, 9,614,640 vaccine doses were administered. A total of 2,732,258 individuals received a first booster dose, corresponding to 78.7% of those with at least one vaccination. The median municipal first booster dose rate (FBDR) was 72.3% [IQR 69.3–75.7] (Table 1).

**Figure 1:**
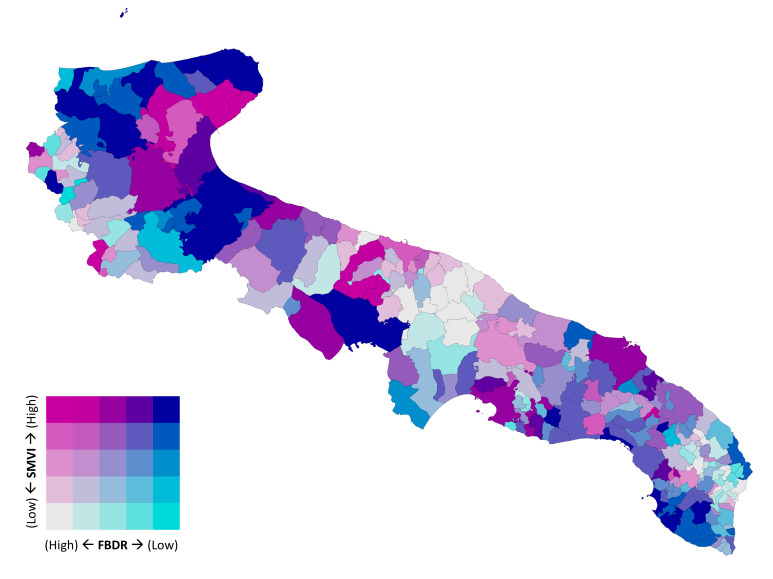
Choropleth map of Apulian municipalities by bivariate distribution highlighting overlap between high deprivation and low booster coverage.

Figure 1 shows a bivariate choropleth map highlighting municipalities with overlapping high deprivation and low booster coverage. Overall, about 15% of municipalities in the highest SMVI quintile also fell into the lowest quintile of booster uptake and 10% of municipalities in the lowest SMVI quintile fell into the highest quintile of booster uptake, underscoring the spatial overlap between socioeconomic disadvantage and low vaccination coverage. The single choropleth maps of SMVI quintiles and FBDR quintiles are shown in Supplementary Figure S2.

In negative binomial models, FBDR was significantly associated with age class (*p* < 0.0001 in both sexes), SMVI class (*p* < 0.0001), COVID-19 infection rate (*p* = 0.0263 in females, *p* = 0.0002 in males), vaccination coverage (≥1 dose) rate (*p* = 0.0005 in females, *p* < 0.0001 in males), and first dose viral vector vaccine rate (*p* = 0.0018 in females, *p* = 0.0008 in males). Model fit indices (AIC and BIC) consistently favored the negative binomial specification over the Poisson model, confirming the presence of overdispersion and supporting the choice of the negative binomial model for inference.

Younger age groups showed significantly lower booster coverage than older adults, with the only non-significant differences being between ages 60–69, 70–79, and ≥80 years in both sexes.

Among females living in municipalities with the highest SMVI (>102), booster coverage was not significantly different compared with the adjacent category (SMVI 101–102), but was significantly lower when compared with lower-deprivation groups (RR = 0.98 [95% CI 0.96–0.99] vs. SMVI 100–101; RR = 0.96 [95% CI 0.95–0.98] vs. SMVI 99–100; RR = 0.95 [95% CI 0.94–0.97] vs. SMVI <99). Similar patterns were observed in males, with slightly stronger associations (RR = 0.97 [95% CI 0.96–0.99] vs. SMVI 100–101; RR = 0.95 [95% CI 0.94–0.97] vs. SMVI 99–100; RR = 0.95 [95% CI 0.93–0.96] vs. SMVI <99).

**Figure 2:**
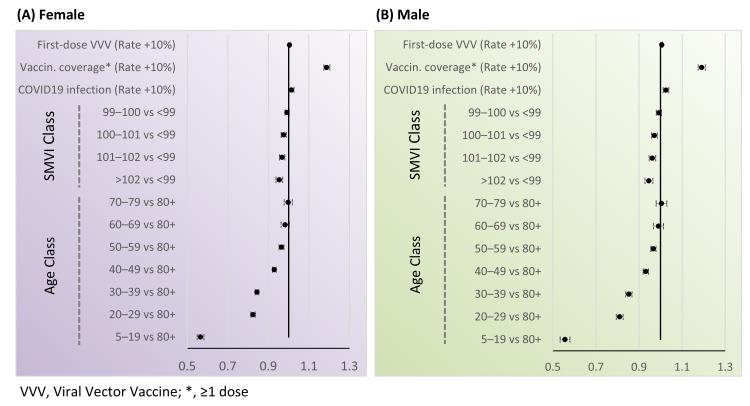
Rate ratios and their 95% confidence interval for first booster dose rate by SMVI class, age class, COVID-19 infection rate, vaccination coverage rate, and first dose viral vector vaccine rate: (**A**) female; (**B**) male.

Higher local infection rates were associated with increased booster uptake (RR for a 10% increase = 1.014 [95% CI 1.001–1.026]). Booster uptake was also positively associated with overall vaccination coverage (RR for a 10% increase = 1.188 [95% CI 1.174–1.203]) and with the first dose viral vector vaccine rate (RR for a 10% increase = 1.004 [95% CI 1.002–1.007]) (Figure 2; all pairwise comparisons are in Supplementary Table S1).

Finally, the interaction between SMVI and age class was significant (*p* = 0.0007 in females, *p* < 0.0001 in males). In both sexes, booster coverage showed an inverse relationship with socioeconomic deprivation, particularly in younger age groups.

Among females, coverage was relatively stable in the 5–19 years group, ranging from 46.0% (95% CI: 44.8–47.3) in the least deprived to 44.9% (95% CI: 43.7–46.1) in the most deprived municipalities, with no significant differences. In contrast, a clear deprivation gradient emerged in the 20–29 years group, where coverage decreased from 69.2% (95% CI: 68.3–70.2) to 62.6% (95% CI: 61.4–63.8), with all pairwise comparisons among the highest deprivation classes being significant (*p* < 0.05). Similar differences were observed in the 30–39 years group (70.0%, 95% CI: 69.0–71.0 vs. 64.7%, 95% CI: 63.5–65.9). The effect was weaker in older women: although coverage decreased slightly with deprivation (e.g., 79.4% vs. 77.0% in the 60–69 years group), several comparisons were not statistically significant.

Among males, the social gradient was steeper. In the 20–29 years group, coverage dropped from 67.9% (95% CI: 66.7–69.1) in the least deprived to 60.2% (95% CI: 58.8–61.7) in the most deprived municipalities, with significant differences across almost all deprivation classes. In the 30–39 years group, coverage decreased from 70.3% (95% CI: 69.3–71.3) to 63.7% (95% CI: 62.4–65.0), and in the 40–49 years group from 75.5% (95% CI: 74.7–76.4) to 70.3% (95% CI: 69.2–71.3), with multiple pairwise contrasts reaching statistical significance. As in females, differences were less pronounced in older men, with uptake remaining close to 79% in all deprivation strata among those aged ≥70 years, and only a few significant comparisons.

**Figure 3:**
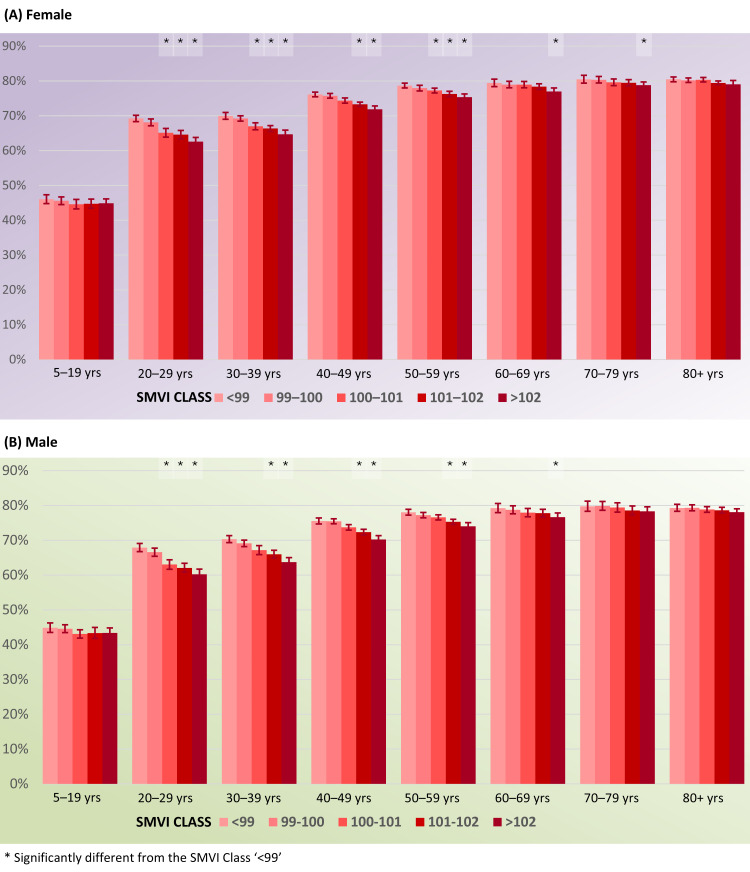
Estimated first booster dose rates and their 95% confident intervals for interaction between SMVI class and age class: (**A**) female; (**B**) male.

Overall, socioeconomic deprivation was consistently associated with lower booster uptake, with the strongest and most statistically robust disparities observed in young and middle-aged males (Figure 3).

The sensitivity analysis confirmed the robustness of the main results in the 190 municipalities with fewer than 15,000 inhabitants, where associations between booster vaccination rates and age class, SMVI, COVID-19 infection rate, vaccination coverage, and viral vector first dose coverage remained statistically significant in both sexes (all *p* < 0.01). In the 52 municipalities with populations between 15,000 and 50,000 inhabitants, all variables remained statistically significant in men, but the COVID-19 infection rate and viral vector first dose coverage were not significant in women. In contrast, among the 15 municipalities with more than 50,000 inhabitants, most associations were attenuated and no longer statistically significant, with the exception of overall vaccination coverage, which remained associated with booster uptake in both females (*p* = 0.0426) and males (*p* = 0.0239). Interaction terms between age class and SMVI were also significant only in smaller municipalities for male gender (*p* = 0.0071 in municipalities with fewer than 15,000 inhabitants and *p* = 0.0015 in municipalities with between 15,000 and 50,000 inhabitants). The full results of the sensitivity analysis are reported in Supplementary Table S2. These findings suggest that the ecological bias due to within-municipality heterogeneity is more likely to affect larger urban areas, whereas in smaller municipalities the SMVI more consistently captures the association with vaccination uptake.

## 4. Discussion

The aim of this study is to evaluate the possible association between the SARS-CoV-2 first booster vaccination rate and social and material deprivation. An analysis of the available data revealed that areas with higher levels of deprivation corresponded to first booster vaccination rates below the regional average. It was also observed that booster uptake was lower among individuals under the age of 50, while no significant difference was found between males and females. An additional finding was that the booster rate was higher in areas with a greater incidence of COVID-19.

Geographic distribution analysis is a highly useful tool for understanding the local epidemiological situation. Numerous studies developed following the COVID-19 pandemic have highlighted how the spatial analysis of events can help assess the spread of the epidemic, as well as the association between case distribution and socioeconomic conditions or the allocation of healthcare resources [34,35]. Although some authors point out that many spatial analyses have focused on mapping and pattern description, socioeconomic data or sufficiently fine-scale data are often lacking to allow robust analyses of causal associations [34]. Implementing surveillance systems that incorporate spatial analysis has also enabled the monitoring of vaccination and vaccine hesitancy rates, allowing for rapid surveys to investigate underlying causes and suggest actions for health authorities to improve vaccination uptake [36,37,38].

The challenge lies in having tools that allow for the rapid use of surveillance data and alternative monitoring systems, alongside current data such as those used in this study, which unfortunately suffer from evident delays in processing. Another difficulty is the need to comply with privacy regulations. For this reason, the use of modern technologies such as data mirroring and replication, or the use of aggregated data at various levels that prevent individual identification, still enables spatial analysis while respecting legal norms and citizens’ rights, and ensuring the operational functionality of public health systems [39,40].

In many studies, the analysis of the socioeconomic context has been associated with vaccination response [20,23]. In our work, we used the SMVI (Social and Material Vulnerability Index), which measures vulnerability based on social factors, such as educational level and family composition, and also considers economic disadvantage indirectly through employment status and family caregiving burden, which contribute to increased health and social expenditures.

The results showed that the higher the vulnerability, the lower the vaccination rate. The results presented originate from an observational study based on aggregated secondary data produced by two distinct entities for the municipal area. Such a design does not permit the inference of a causal relationship between the two primary variables under examination; nevertheless, it invites a consideration of the implications of a potential association. In the study by Lee et al. [41], it is clearly stated that low educational levels and certain political beliefs are associated with lower vaccination rates; living in rural areas compromises vaccination uptake due to limited access to services. Another study in a different context confirmed the impact of education on vaccination decisions and highlighted that the effect is more pronounced among women [42]. Again, rural residence is an important factor that may reduce vaccine acceptance [43]. These two studies gave, therefore, a confirmation of the association between social conditions and vaccine hesitancy with individual data analysis.

The study by Roy [44] analyzed individual social determinants and confirmed the influence of education, while also showing the positive effect of being part of a household and having a higher income.

An analysis with individual data often evaluates social determinants with individual conditions, while population analysis determines the use of synthetic indexes. Using a single index that summarizes all these aspects allows for a comprehensive evaluation and can be a useful tool for health decision-makers who prefer concise and meaningful reports. The SMVI encompasses all the previously described factors, and our analysis suggests that the Apulian regional context aligns with findings from other studies [45,46]. Moreover, the use of this index is consistent and does not alter the direction of the association, even though it aggregates different sub-indicators that could interfere with direct relationships.

There is another example of analysis at the population level that has explored the association of social determinants and vaccine hesitancy, and the results are similar to those reported in our paper. A report on the socioeconomic determinants of vaccine uptake [47] linked both education level and household income to higher vaccination rates. However, that report also used a synthetic index of socioeconomic status (SEIFA-IRSD) and found an association between high social deprivation and low vaccine acceptance.

A 2023 review [46] examined the association between low vaccination rates and socioeconomic status described by synthetic indices similar in structure to the one used in our analysis. The review identified a consistent association between high deprivation and low vaccination rates.

We can conclude that both types of studies analyzing area-level deprivation indices and those examining individual determinants—many of which are components of the synthetic indices—have shown an association between disadvantaged conditions and lower vaccination rates, as observed in our analysis. Furthermore, the use of area-level synthetic indices did not alter the direction of the association.

In our model, then, we have adjusted the analysis accounting for COVID-19 rates, because we thought that higher rates could have driven more people to vaccination. The results have shown effectively that there was a positive association between disease rate and vaccination coverage, but this did not change the direction of the association between vaccine coverage and social deprivation.

Our results did not show a gender-related effect, which was actually expected, especially considering that in the cited studies, female subjects consistently reported higher vaccination rates. The study by Morales [48] demonstrated the impact of socioeconomic determinants by evaluating gender disparities: women who work or live in poverty were more hesitant compared with men. Nevertheless, in other studies, higher educational attainment among women appears to be associated with higher vaccination rates, although these studies did not directly compare the effect with men [42,49].

In our analysis, however, gender does not appear to be associated with lower vaccination rates. This association was anticipated, particularly because the SMVI includes variables such as single parenthood with children and caregiving responsibilities for elderly individuals—factors that often fall more heavily on women than men. Therefore, a stronger gender effect could have been expected.

One possible explanation for the lack of association may be that, in our context [Apulia], vaccination uptake was indeed similar between males and females, and there may be no association between gender and deprivation—although this cannot be demonstrated with the aggregated data available to us. Nevertheless, vaccination adherence was higher in women in many studies [44,46,47], but there is not an analysis of interaction to evaluate if there are changes in more deprived levels.

The association between higher vaccination uptake and areas with greater COVID-19 incidence is also noteworthy. Since this association was observed in an analysis of aggregated municipal-level data, we believe that risk perception and prior experience with infection—knowing that it does not confer permanent immunity—may have facilitated the decision to get vaccinated and positively influenced booster dose acceptance.

A 2023 study [50] found that individuals who had previously contracted COVID-19, especially those over 55 years of age, were more likely to accept the booster dose. Another analysis, however, showed a lower tendency to receive the bivalent booster dose, with individuals preferring to wait and observe infection trends before deciding [51].

A 2022 review summarized that COVID-19 vaccine acceptance factors are linked to pandemic-related risk perception, trust in healthcare workers, and previous experiences with other vaccines, particularly regarding safety [52]. Further insights into vaccine hesitancy were provided by Krasner [53], who used a composite index and showed that, at a macro-area level [not individual], higher distress due to illness, risk, and disadvantaged social conditions influenced vaccine acceptance.

Recent studies [54,55] have further explored vaccine intentions among young adults and across different population segments, confirming the influence of socioeconomic and psychological factors.

Area-level analysis involves an approximation that aggregates a diverse population into a single view, with the risk of capturing trends that may not be realistic. The sensitivity analysis included in our results helps to moderate confidence or skepticism regarding the findings. The results suggest that for medium-to-small municipalities—which represent the majority of territorial communities—the analysis is reliable, whereas for larger municipalities, the associations are less dependable. This highlights the need to conduct small area analyses in areas exceeding 50,000 inhabitants.

The consistency of our observations with the available research conducted through individual questionnaires, as well as with other studies that considered macro-area indicators, allows us to conclude that mapping indicators of social disadvantage alongside vaccination response indicators enables the identification of target groups for appropriate informational and persuasive campaigns. These efforts aim to tailor communication strategies and achieve the best possible outcomes in terms of vaccination coverage, which remains the most effective tool to counter high infectious disease transmission.

It is worth noting that the analytical method used can be extended to other conditions, particularly to the spatial distribution analysis of influenza vaccination rates. Understanding the socioeconomic context allows for the implementation of effective communication strategies to improve adherence to vaccination campaigns. The success of such campaigns relies on trust in healthcare personnel and on providing adequate information regarding risks and benefits [8,56,57].

However, several limitations of this analysis should be acknowledged. First, the reliance on aggregated data. While this approach facilitates the assessment of epidemiological and social conditions without requiring resource-intensive, sample-specific investigations and enables the use of timely data to inform strategic actions, it also introduces the risk of ecological bias—where observed associations may not accurately reflect individual-level variability. Although sensitivity analyses were conducted to mitigate this issue, it remains an inherent limitation.

A further concern relates to the use of composite indices, which do not allow for the disentanglement of individual components underlying social deprivation. Consequently, while an overall association between socioeconomic deprivation and attitudes toward vaccination can be identified, these indices cannot reveal which specific dimension of deprivation exerts the greatest influence on vaccination decisions. Moreover, although this study focused on individuals aged over five years, it is important to note that vaccination choices among children and younger adolescents are rarely autonomous and are strongly shaped by family practices. Unfortunately, aggregated socioeconomic indicators do not permit this additional level of analysis, which would be crucial to understanding the role of parental or guardian beliefs in shaping vaccination behaviors among individuals under 18 years of age. Secondly, the reliability of vaccination coverage data must also be considered. We trust the data we have used because in that period there was strong attention to collect any event about the disease and vaccination, since there was a military force that supervised the data flow; however, we cannot completely exclude an underestimation of vaccine rate. This could affect our analysis for another reason: the algorithm used to find and aggregate subjects could have excluded people and could have led to underestimation.

Another limitation is the different timing of data publication and access, a problem also highlighted in the work by Biddle et al. [58]. The SMVI dates back to 2011, while the epidemic data are from 2021. Although updated data on infection spread and vaccination coverage are currently accessible, the SMVI has not yet been recalculated. Our research group are studying the bias that could happen in these studies of association using available official sources. Our aim is to evaluate the longitudinal validity of the SMVI categorized into quartiles, and we have observed that the value of the index of each municipality generally maintained their relative position over time compared with the quartiles defined at the time of data collection [59]. Overall, we cannot exclude bias in the measurement of social inequalities and we would like a faster update of this area-level index, and we hope that the evolution of digital applications will help overcome this issue.

## 5. Conclusions

This study identified an association between socioeconomic disadvantage and reduced uptake of the first booster dose of the COVID-19 vaccine. This decline was especially notable for people under 50 years of age and who were highly vulnerable, while no notable differences were observed between genders.

The results indicate that certain population subgroups—defined by age and vulnerability—could benefit from tailored communication strategies designed to enhance awareness and address vaccine hesitancy. This last conclusion, which is related to the subgroups of populations, is mainly due to the following limitation of the research: its reliance on aggregated data, which determines an ecological fallacy, which does not allow detailed analysis at the individual level and may result in overgeneralized conclusions. More accurate studies at the individual level with a robust methodology for sampling could help to evaluate the relation of vulnerability with awareness in the decision to accept a vaccine.

## Figures and Tables

**Table 1:** Characteristics of the study population and municipal-level indicators (Apulia region, 1 January 2021–31 December 2022).

Characteristic	Value
Individuals with ≥1 COVID-19 vaccine dose	3,472,095
Age (years)	Range: 5–110
Sex	Female: 51.6%; Male: 48.4%
Total vaccine doses administered	9,614,640
Individuals receiving ≥1 booster dose	2,732,258
First booster dose rate (FBDR)	78.7% among individuals with ≥1 dose
Municipalities included	257
Median municipal FBDR [IQR]	72.3% [69.3–75.7%]

## Data Availability

The data for the analysis were sourced directly from the Health Information System of the Apulia Region and are not publicly available due to the nature of the information and due to privacy restrictions.

## Supplementary Materials

The following supporting information can be downloaded at: https://www.mdpi.com/article/10.3390/vaccines13121255/s1, Figure S1. Algorithm used to identify the first booster dose from individual vaccination histories. Figure S2. Choropleth maps of Apulian municipalities: (A) by quintiles of the Social and Material Vulnerability Index (SMVI); (B) by quintiles of the first booster dose rate (FBDR). Table S1. Rate ratios and their 95% confidence interval for first booster dose rate by SMVI class, age class, COVID-19 infection rate, vaccination coverage rate, and first dose viral vector vaccine rate, by gender. Table S2: Sensitivity analysis of negative binomial regression models: comparison of *p*-values for model parameters estimated using all municipalities, municipalities with <50,000 inhabitants, and municipalities with ≥50,000 inhabitants in Apulia, Italy, 2021–2022.

## Abbreviations

The following abbreviations are used in this manuscript:

ISTAT	Italian Institute of Statistics
SMVI	Social and Material Vulnerability Index
