# Supplementary material for: Socioeconomic Barriers to COVID-19 Booster Vaccination in Southern Italy: A Retrospective Study to Evaluate Association with the Social and Material Vulnerability Index in Apulia
Source: Vaccines (Basel). 2025 Dec 18;13(12):1255. doi: 10.3390/vaccines13121255 (PMC12737561; doi:10.3390/vaccines13121255)
Supplement: Supplementary file 1 [file vaccines-13-01255-s001.zip › vaccines-3934032-supplementary.pdf]

Supplementary Materials

Algorithm for identifying the First Booster Dose

In the regional vaccination registry, no variable explicitly identified whether an administered dose was a first booster dose. However, for each individual, a complete chronological record of all COVID-19 vaccine administrations was available, including:
(1) the nominal dose number (e.g., “first dose”, “second dose”, “third dose”),
(2) the administration date,
(3) the vaccine product.

Because the booster dose does not necessarily coincide with the nominal “third dose”—for example, individuals vaccinated with Janssen received a booster after a single-dose primary cycle—the first booster dose was reconstructed algorithmically. The algorithm also accounted for potential SARS-CoV-2 infection before or after the primary cycle, which could modify the recommended booster schedule according to national guidelines. Below we detail the rules applied.

1. Individuals with two or more recorded doses with coherent numbering
(e.g., “first dose” → “second dose” → “third dose”, etc., or starting from “second dose” → “third dose” → “fourth dose”, etc.)

For these individuals, the first booster dose was identified using the interval between two consecutive administrations ($DS_x - DS_{x-1}$), applying the minimum recommended time gaps established in the Ministry of Health circulars.
The logic is illustrated in Supplementary Figure S1, where S_x denotes the x-th administration and D the administration date.

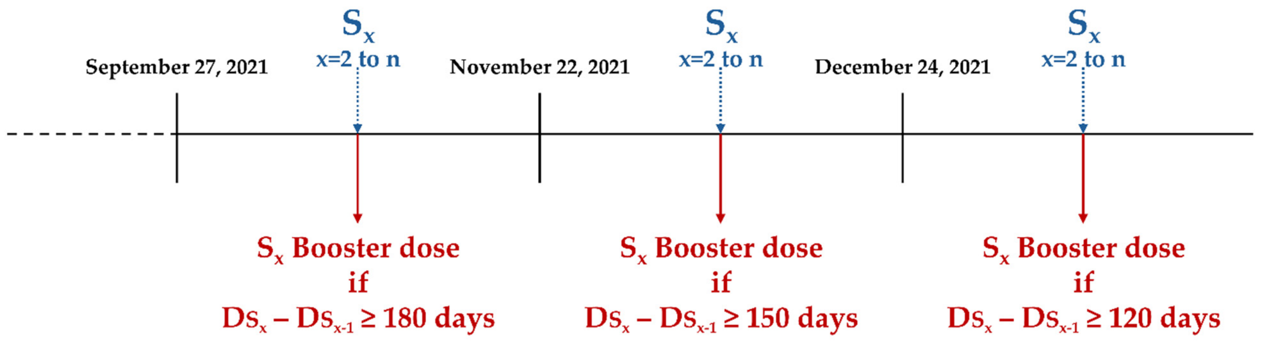

Figure S1. Algorithm used to identify the first booster dose from individual vaccination histories.

A dose S_x was classified as the first booster if the interval from the preceding dose met the following criteria:
≥180 days from the previous dose (initial recommendation period), or
≥150 days (first revision), or
≥120 days (latest revision during the study period).
Only the earliest dose satisfying one of these time thresholds was classified as the first booster dose.

2. Individuals with inconsistent nominal dose numbering
(e.g., “first dose” → “third dose”, “first dose” → “fourth dose”, etc.)

For individuals whose second recorded administration was labeled as a “third” or “fourth” dose, the second administration was automatically classified as the first booster dose, as such patterns typically occur when the second dose corresponds to the first booster after a single-dose Janssen primary cycle or following prior SARS-CoV-2 infection.

3. Individuals whose primary cycle was completed outside the region
(e.g., first available record is “third dose”, possibly followed by “fourth dose”)

For subjects whose earliest registered dose was labeled as “third dose” (and subsequent doses as “fourth dose”, etc.), the first recorded administration was considered the first booster dose if administered on or after 27 September 2021, the official start date of the booster vaccination campaign in Italy.

Notes on data completeness and consistency
Sex, age, municipality of residence, and all dates of administration are mandatory fields in the regional vaccination registry.
Therefore, the algorithm always had complete information for dose reconstruction, and chronological inconsistencies (e.g., a "third dose" dated before a "second dose") were resolved automatically through the time-interval rules described above.

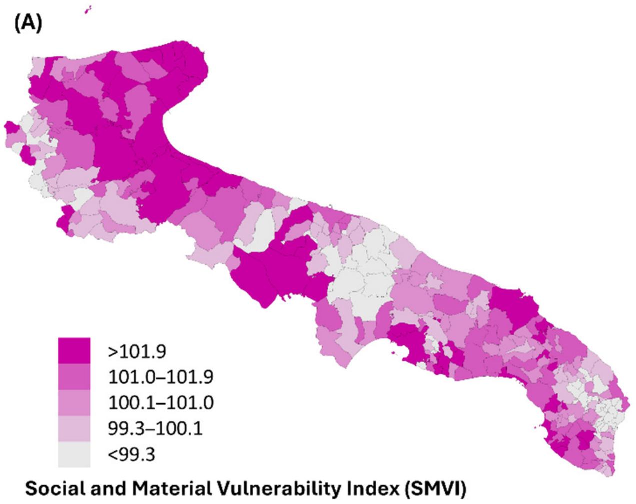

Figure S2. Choropleth maps of Apulian municipalities: (A) by quintiles of the Social and Material Vulnerability Index (SMVI); (B) by quintiles of the first booster dose rate (FBDR).

Table S1. Rate ratios and their 95% confidence interval for First Booster Dose Rate by SMVI class, age class, COVID19 infection rate, Vaccination coverage rate and first-dose viral vector vaccine rate, by gender.

Covariate	Comparison	Rate Ratio [95% CI]
Age Class	5–19 vs 20–29	0.69 [0.67–0.71]
	5–19 vs 30–39	0.67 [0.65–0.69]
	5–19 vs 40–49	0.61 [0.59–0.63]
	5–19 vs 50–59	0.59 [0.56–0.61]
	5–19 vs 60–69	0.58 [0.55–0.60]
	5–19 vs 70–79	0.57 [0.54–0.59]
	5–19 vs 80+	0.57 [0.55–0.58]
	20–29 vs 30–39	0.98 [0.97–0.99]
	20–29 vs 40–49	0.89 [0.88–0.90]
	20–29 vs 50–59	0.85 [0.84–0.87]
	20–29 vs 60–69	0.84 [0.82–0.86]
	20–29 vs 70–79	0.83 [0.81–0.85]
	20–29 vs 80+	0.82 [0.81–0.84]
	30–39 vs 40–49	0.91 [0.90–0.91]
	30–39 vs 50–59	0.87 [0.86–0.88]
	30–39 vs 60–69	0.86 [0.84–0.88]
	30–39 vs 70–79	0.85 [0.83–0.86]
	30–39 vs 80+	0.84 [0.83–0.85]
	40–49 vs 50–59	0.96 [0.96–0.97]
	40–49 vs 60–69	0.95 [0.93–0.96]
	40–49 vs 70–79	0.93 [0.92–0.95]
	40–49 vs 80+	0.93 [0.92–0.94]
	50–59 vs 60–69	0.98 [0.97–0.99]
	50–59 vs 70–79	0.97 [0.95–0.98]
	50–59 vs 80+	0.96 [0.95–0.98]
	60–69 vs 70–79	0.98 [0.98–0.99]
	60–69 vs 80+	0.98 [0.96–1.00]
	70–79 vs 80+	1.00 [0.98–1.02]
SMVI Class	>102 vs 101–102	0.99 [0.97–1.00]
	>102 vs 100–101	0.98 [0.96–1.00]
	>102 vs 99–100	0.96 [0.95–0.98]
	>102 vs <99	0.95 [0.94–0.97]
	101–102 vs 100–101	0.99 [0.98–1.01]
	101–102 vs 99–100	0.98 [0.96–0.99]
	101–102 vs <99	0.97 [0.96–0.98]
	100–101 vs 99–100	0.98 [0.97–1.00]
	100–101 vs <99	0.98 [0.96–0.99]
	99–100 vs <99	0.99 [0.98–1.00]
COVID19 infection	Rate +10%	1.014 [1.001–1.026]
Vaccin. coverage*	Rate +10%	1.188 [1.174–1.203]
First-dose VVV	Rate +10%	1.004 [1.002–1.007]

The Table S2 reports *p*-values from negative binomial regression models assessing the association between first booster dose rate and model covariates (age class, SMVI class, COVID-19 infection rate, vaccination coverage, and viral vector first-dose rate). Results are shown for models including all municipalities, municipalities with <50,000 inhabitants, and municipalities with ≥50,000 inhabitants. Stratification by municipality size was performed as sensitivity analysis to assess potential ecological bias in the use of SMVI.

Table S2. Sensitivity analysis of negative binomial regression models: comparison of *p*-values for model parameters estimated using all municipalities, municipalities with <15,000 inhabitants, municipalities with inhabitants between 15,000 and 50,000 and municipalities with ≥50,000 inhabitants in Apulia, Italy, 2021–2022.

Sex	Parameter	Municipalities			
		All (n = 257)	<15,000 inhabitants (n = 190)	15.000–50,000 inhabitants (n = 52)	50,000+ inhabitants (n = 15)
Female	Age group	**<0.0001**	**<0.0001**	**<0.0001**	0.2894
	SMVI group	**<0.0001**	**<0.0001**	**<0.0001**	0.5467
	COVID19 infection rate	**0.0263**	**0.0132**	0.5213	0.1785
	Vaccination Coverage Rate (≥1 dose)	**0.0005**	**0.0133**	**<0001**	**0.0426**
	First-dose VVV Rate	**0.0018**	**0.0053**	0.0569	0.1752
	Age group*SMVI group	**0.0007**	0.0908	0.1906	0.4066
Male	Age group	**<0.0001**	**<0.0001**	**<0.0001**	0.0738
	SMVI group	**<0.0001**	**<0.0001**	**<0.0001**	0.6624
	COVID19 infection rate	**0.0002**	**0.0291**	**0.0323**	0.3015
	Vaccination Coverage Rate (≥1 dose)	**<0.0001**	**0.0035**	**<0.0001**	**0.0239**
	First-dose VVV Rate	**0.0008**	**0.0071**	**0.0015**	0.7041
	Age group*SMVI group	**<0.0001**	**0.0177**	**0.0450**	0.3974

VVV, Viral Vector Vaccine
